# 3D-Printed Patient-Specific Instrumentation Versus Conventional Techniques in Total Knee Arthroplasty: A Systematic Review and Meta-Analysis

**DOI:** 10.7759/cureus.94626

**Published:** 2025-10-15

**Authors:** Abdelfatah M Elsenosy, Eslam Hassan, Ahmed S Yousef, Mustafa Al-Alawi

**Affiliations:** 1 Trauma and Orthopedics, Frimley Park Hospital, Frimley, GBR; 2 Trauma and Orthopedics, Poole General Hospital, Poole, GBR; 3 Trauma and Orthopedics, University Hospitals Dorset, Poole, GBR; 4 Emergency Medicine, Frimley Park Hospital, Frimley, GBR

**Keywords:** 3d printing, alignment accuracy, meta-analysis, patient-specific instrumentation, surgical outcomes, total knee arthroplasty

## Abstract

3D-printed patient-specific instrumentation (PSI) in total knee arthroplasty (TKA) has been proposed to improve surgical precision and outcomes compared to conventional instrumentation (CI), but its clinical benefits remain uncertain. We conducted a systematic review and meta-analysis of 14 comparative studies, including 2,704 TKA procedures, evaluating surgical time, intraoperative blood loss, alignment accuracy, and malalignment rates. Databases searched included PubMed, Embase, Scopus, Web of Science, and Cochrane CENTRAL. Random-effects meta-analysis demonstrated that PSI significantly reduced hip-knee-ankle alignment outliers (OR = 0.30, p < 0.00001), improved overall alignment accuracy (standardized mean difference (SMD) = -0.27, p = 0.02), and decreased intraoperative blood loss (SMD = -1.05, p = 0.009), while no significant difference was observed in surgical time (SMD = -0.78, p = 0.17), despite high heterogeneity. Complication rates were similar between groups. These findings indicate that 3D-printed PSI enhances alignment precision and reduces blood loss in TKA without increasing perioperative risk, highlighting its potential utility in complex cases or high-volume surgical settings. However, these results should be interpreted with caution given the predominance of observational studies and generally short follow-up durations among the included literature. Further high-quality trials are warranted to clarify its long-term clinical, functional, and economic impact.

## Introduction and background

Total knee arthroplasty (TKA), also known as total knee replacement, is a surgical intervention designed to relieve pain and restore function in patients with severe knee joint damage, most commonly due to osteoarthritis, rheumatoid arthritis, or post-traumatic arthritis. TKA is now one of the most commonly performed orthopedic procedures worldwide, with over 600,000 surgeries annually in the United States alone [1]. The goal of TKA is to alleviate pain, improve joint function, and enhance overall quality of life by replacing the damaged articular surfaces of the knee with artificial components, which typically include implants for the femur, tibia, and patella. Advances in implant design, surgical techniques, and perioperative care have significantly improved the success rates and longevity of TKA over the last few decades [2].

Despite these advancements, the procedure carries potential risks, including infection, blood clots, prosthetic loosening, and improper alignment. Precision in component placement is critical, as malalignment can lead to early failure and revision surgery. This challenge has prompted the development of computer-assisted and robotic-assisted surgical methods to enhance alignment accuracy and functional outcomes [3].

Patient-specific instrumentation (PSI) has emerged over the past decade as a promising innovation in TKA, offering a more individualized surgical approach. PSI involves the use of patient imaging data, typically from MRI or CT scans, to design custom cutting guides tailored to each patient’s unique anatomy. These patient-specific guides replicate the planned bone resections and alignment angles derived from preoperative 3D models, enabling accurate component placement without reliance on intramedullary alignment rods. This individualized approach directly addresses key limitations of conventional jigs, such as dependence on intramedullary referencing and limited visualization in knees with deformity, bone loss, or extra-articular bowing. By conforming precisely to each patient’s osseous contours, PSI minimizes alignment errors caused by canal distortion, osteophyte formation, or restricted visualization. This improved anatomical fit enhances guide stability and helps achieve more consistent coronal, sagittal, and rotational alignment. Importantly, these advantages are particularly relevant in complex deformities, post-traumatic knees, or cases where conventional jigs may be less reliable and alignment more difficult to reproduce [4,5].

The theoretical benefits of PSI include better component alignment and a reduction in radiographic outliers, potentially leading to improved implant longevity. Some clinical studies have shown modest improvements in alignment and reductions in surgical time and blood loss with PSI compared to conventional methods [6,7]. In high-volume centers, efficiency gains from reduced instrumentation and faster setup have even offset the added costs of preoperative imaging and guide production [8].

However, the clinical advantages of PSI remain debated. Large-scale randomized controlled trials have found no significant differences in long-term functional outcomes or implant alignment between PSI and conventional techniques [9,10]. Concerns also remain regarding cost-effectiveness, particularly in lower-volume hospitals where efficiency gains may not justify the added costs [11].

3D printing has played an increasingly important role in enhancing surgical precision and personalization in TKA. By using advanced imaging techniques like CT and MRI, 3D printing enables the creation of PSI, customized cutting guides, and even personalized implants that conform precisely to an individual’s anatomy. This technology improves preoperative planning and allows for highly accurate bone resections and component placements during surgery [12,13]. Clinical studies have demonstrated that 3D printing can enhance precision in component alignment and surgical execution, reduce operative time, and minimize blood loss compared to conventional methods. For example, patients undergoing TKA with 3D-printed guidance experienced significantly improved alignment and reduced intraoperative bleeding and postoperative drainage compared to conventional techniques [14,15]. These findings highlight the practical clinical relevance of PSI and 3D printing technologies, particularly in anatomically complex knees where precision and reproducibility are essential for optimal outcomes.

Moreover, 3D printing is especially valuable in complex revision surgeries or cases involving bone defects. The creation of custom porous tantalum implants and spacers has enabled surgeons to restore bone integrity and joint stability in challenging cases, with studies reporting significant improvements in function and pain reduction [16,17]. Beyond direct surgical benefits, 3D printing has transformed surgical education by allowing trainees to practice procedures on anatomically accurate models, improving their skills in a low-risk environment [18].

Recent comparative studies highlight both the clinical advantages and limitations of 3D-printed PSI (3D-PSI) compared to conventional techniques in TKA. Evidence shows that 3D-PSI enhances surgical precision and alignment, with patients demonstrating significantly lower deviation from target hip-knee-ankle (HKA) angles and fewer alignment outliers [19-21]. PSI has also been associated with reduced surgical time and less intraoperative blood loss, contributing to improved perioperative efficiency [22,23]. In terms of early postoperative outcomes, several studies report comparable functional recovery between PSI and conventional techniques. While some found improved Knee Society Scores (KSSs) or reduced pain in PSI groups during early follow-up [15], others observed no significant long-term functional differences [9,10,15]. Long-term studies confirm PSI’s radiological benefits but challenge its superiority in overall implant survival and patient-reported outcomes.

Objective

This systematic review and meta-analysis aims to evaluate the clinical efficacy and surgical precision of 3D-PSI compared to conventional instrumentation (CI) in primary TKA.

## Review

Methods

Search Strategy

A comprehensive literature search was conducted to identify studies comparing 3D-PSI with CI in primary TKA. Five databases, such as PubMed (MEDLINE), Embase, Scopus, Web of Science, and the Cochrane Central Register of Controlled Trials, were systematically searched from inception to May 2025, with a restriction to studies published in the last 15 years.

The search combined Medical Subject Headings (MeSH) and relevant free-text keywords, including “patient-specific instrumentation”, “3D printed cutting blocks”, “custom guides”, and “total knee arthroplasty”. Boolean operators were applied to link terms describing both the procedure and the intervention. Additionally, reference lists of eligible studies and relevant reviews were manually screened to ensure the inclusion of all pertinent literature.

Inclusion and Exclusion Criteria

Both randomized and non-randomized comparative studies were eligible for inclusion, provided they reported at least one relevant perioperative or radiological outcome. Methodological quality was assessed using the modified Downs and Black checklist, and only studies rated as fair to excellent were included in the final analysis. Studies categorized as poor (score ≤14) were excluded to maintain an acceptable overall level of methodological quality. The eligibility criteria applied during the study selection process are summarized in Table 1, outlining both the inclusion and exclusion criteria.

**Table 1:** Inclusion and exclusion criteria for studies in the systematic review HKA, hip-knee-ankle; PSI, patient-specific instrumentation; TKA, total knee arthroplasty

Inclusion criteria	Exclusion criteria
Adult patients (≥18 years) undergoing primary TKA	Review articles, case reports, editorials, technical notes, cadaveric or biomechanical studies, or abstracts without full text
Direct comparison of 3D-printed or imaging-based PSI vs conventional intramedullary or extramedullary alignment guides	Studies focusing solely on revision surgeries
Reported at least one measurable perioperative or radiological outcome (e.g., surgical time, intraoperative blood loss, HKA alignment, and malalignment rates)	Studies without a direct comparator group

Outcome Measures

The primary outcomes of interest were surgical time (minutes), intraoperative blood loss (milliliters), HKA alignment accuracy (absolute deviation in degrees from neutral alignment), and alignment outlier rate (deviation > ±3° from the mechanical axis). Where data were available, additional outcomes such as tourniquet time, early postoperative complications, and functional scores (e.g., KSS) were also considered for descriptive synthesis.

Data Extraction and Quality Assessment

Two independent reviewers extracted data on study characteristics, including author, year of publication, country, study design, sample size, patient demographics, imaging and manufacturing methods for 3D-PSI, and follow-up duration. Outcome data collected included means and standard deviations for continuous variables and event counts for binary variables. Discrepancies between reviewers during data extraction or quality assessment were resolved through discussion and consensus, with arbitration by a third reviewer when necessary. The methodological quality of each study was assessed using the modified Downs and Black checklist, which evaluates reporting clarity, external validity, internal validity (bias and confounding), and statistical power. Studies were categorized as Excellent (score ≥26), Good (21-25), Fair (15-20), or Poor (≤14). Studies rated as poor were excluded from the final analysis due to insufficient methodological quality.

Statistical Analysis

Meta-analyses were conducted using Review Manager (RevMan) version 5.4. For continuous outcomes, standardized mean differences (SMDs) with 95% CIs were calculated, while dichotomous outcomes were pooled using ORs with 95% CIs. A mixed-modeling approach was applied: random-effects models (DerSimonian-Laird method) were used by default to account for expected clinical and methodological heterogeneity, and fixed-effect models were applied when heterogeneity was negligible. Between-study heterogeneity was assessed using the chi-square (Q) test and the I² statistic, with I² values above 50% indicating moderate-to-high heterogeneity. Funnel plots were generated to evaluate potential publication bias, and Egger’s regression test was used for statistical confirmation, with a two-tailed p-value < 0.05 considered indicative of significant bias. All statistical analyses were two-sided, and p-values <0.05 were deemed statistically significant. Missing data were handled by excluding incomplete outcome sets from quantitative synthesis while retaining them for qualitative review where appropriate. The decision to apply fixed- or random-effects models was guided by the level of heterogeneity, with random-effects used when I² exceeded 50% or when clinical or methodological variability was anticipated, ensuring consistent and robust interpretation of pooled results.

Results

Search and Study Selection

A comprehensive systematic search was conducted to identify studies comparing PSI with conventional alignment guides in primary TKA. Databases searched included MEDLINE (via PubMed), Embase, Scopus, Web of Science, and the Cochrane CENTRAL Register, covering records from inception until 2025. The search strategy combined free-text keywords and controlled vocabulary for concepts such as “patient-specific”, “custom cutting block”, “3D-printed”, and “total knee arthroplasty”, along with terms for “alignment”, “instrumentation”, and “cutting guide”. Reference lists of relevant reviews and included studies were manually screened, and content experts were consulted to ensure comprehensiveness.

**Figure 1:**
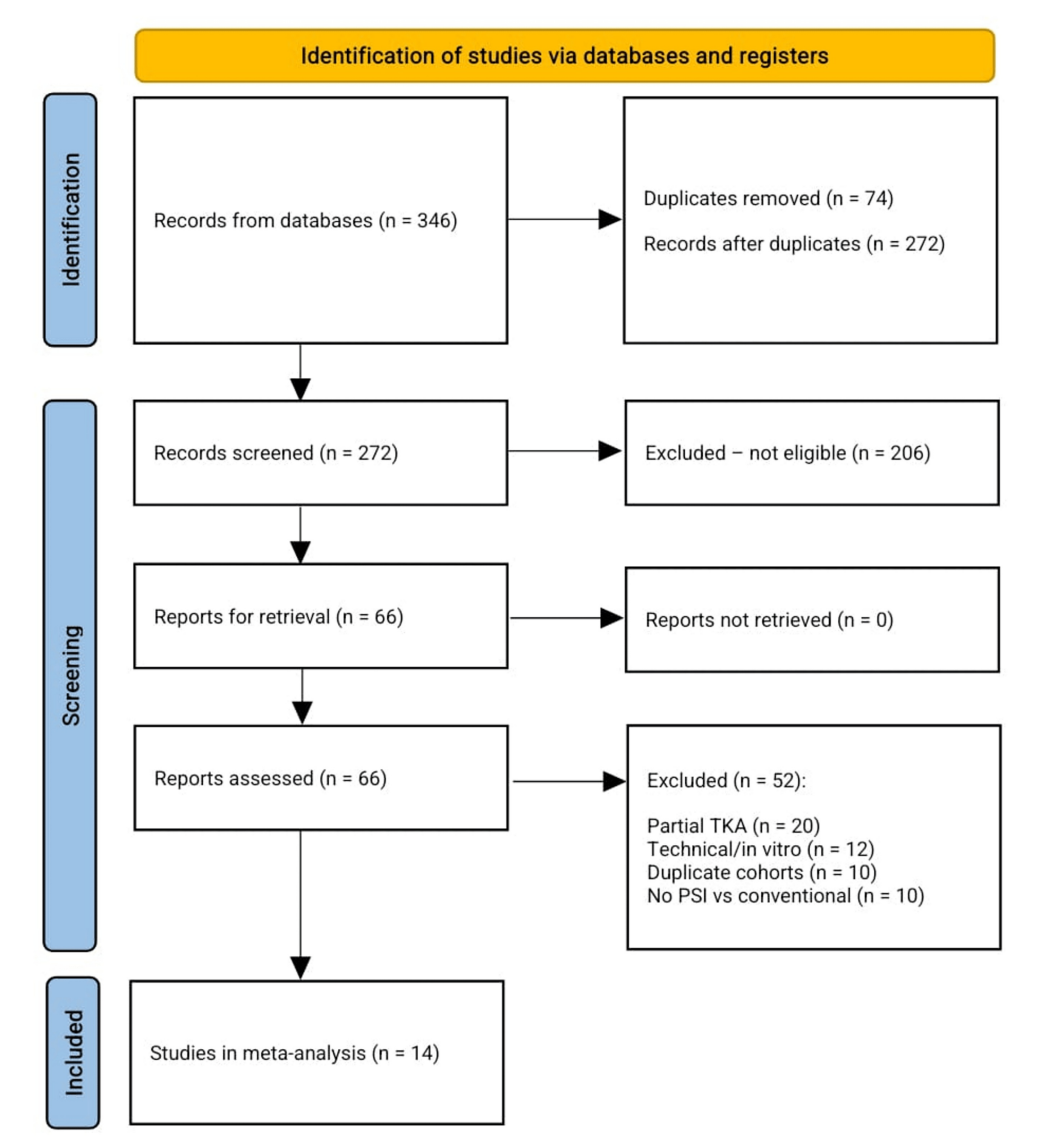
PRISMA flow chart for the included studies The diagram shows the number of records identified through database and manual searches, duplicates removed, records screened, full-text articles assessed for eligibility, and studies included in the qualitative and quantitative analyses. PRISMA, Preferred Reporting Items for Systematic reviews and Meta-Analyses

The initial electronic search yielded 346 records, of which 74 duplicates were removed. Titles and abstracts of the remaining 272 citations were screened against predefined eligibility criteria: (1) adult patients undergoing primary TKA; (2) direct comparison of CT- or MRI-based PSI versus conventional intramedullary femoral and extramedullary tibial guides; and (3) reporting at least one radiological or clinical outcome. After excluding 206 studies for irrelevance or non-comparative design, 66 full texts were assessed. A further 52 articles were excluded due to revision procedures, purely technical reports without outcome data, or overlapping cohorts. Ultimately, 14 studies met all inclusion criteria and were included in the qualitative synthesis and, where possible, quantitative meta-analysis. The detailed study selection process is illustrated in the Preferred Reporting Items for Systematic reviews and Meta-Analyses (PRISMA) flow diagram (Figure 1).

Study Characteristics

This review includes 14 comparative studies, four randomized controlled trials and ten observational cohorts, encompassing a total of 2,704 primary total knee arthroplasties conducted in centers across North America, Europe, and Asia. Participants were predominantly adults in their late sixties (mean age 61-73 years), with a slight female majority (~60%) and body mass indices of 25-33 kg/m², representing the typical osteoarthritic population undergoing knee replacement.

All studies compared CT- or MRI-based 3D-PSI systems (e.g., Visionaire™, MyKnee®, TruMatch™, Prophecy™, or in-house/AI-generated guides) with CI using standard intramedullary femoral and extramedullary tibial alignment jigs provided by the same implant manufacturer. PSI templates were prepared one to three weeks preoperatively; six trials used single-use guides, while the remaining studies used reusable cutting blocks machined for each patient.

Follow-up intervals ranged from immediate postoperative imaging to over two years, although most studies reported radiographic accuracy within the first two weeks and early clinical outcomes at six or 12 months. Core outcomes included limb and component alignment (HKA angle and three-plane component angles), operative metrics (skin-to-skin time, tourniquet time, blood loss, tray count, and theater turnover), standard knee scores (KSS, HSS, WOMAC, and OKS), and safety endpoints such as infection, wound complications, transfusion, or revision surgery.

Across studies, PSI consistently reduced malalignment outlier rates and improved tibio-femoral coronal accuracy compared with conventional jigs. Several randomized trials also demonstrated reductions in blood loss and instrument trays, and in some cases, shorter operating times and faster early recovery, whereas other series observed no operative-time advantage. Complication rates, including infections and early revisions, were generally comparable between groups. The main trade-off for PSI was additional preoperative planning and manufacturing time. Table 2 summarizes each study’s design, sample size, demographic profile, imaging modality, guide manufacturer, follow-up duration, complication rates, and key radiological and clinical outcomes.

**Table 2:** Data extraction for the studies comparing PSI with conventional alignment guides in primary TKA AKS, American Knee Society score; ASA, American Society of Anesthesiologists; CI, conventional instrumentation; CON, conventional group; CVI, conventional instrumentation group; FCR, femoral component rotation; FCA, femoral coronal angle; FFC, femoral flexion component; FMAA, femoral mechanical axis angle; FSA, femoral sagittal angle; HKA, hip-knee-ankle angle; Hb, hemoglobin; HSS, Hospital for Special Surgery score; IM, intramedullary; KSS, Knee Society Score; LFC, lateral femoral component; LTC, lateral tibial component; LOS, length of stay; MME, morphine milligram equivalents; NEW-KSS, New Knee Society Score; OKS, Oxford Knee Score; PCA, posterior condylar angle; PFA, patellar facet angle; POD, postoperative day; PSI, patient-specific instrumentation; PSG, patient-specific guide; RCT, randomized controlled trial; ROM, range of motion; TCA, tibial coronal angle; TSA, tibial sagittal angle; TKA, total knee arthroplasty; VAS, Visual Analogue Scale; WOMAC, Western Ontario & McMaster Universities Osteoarthritis Index

Study	Study design	Sample size	Level of evidence	Patient demographics	Intervention details	Follow-up	Outcome measures	Results	Complications	Conclusions
Anderl et al. [19]	Prospective single-center comparative cohort (CT-based PSI vs CI in mobile-bearing primary TKA)	222 knees analyzed (CVI = 108; PSI = 114) out of 300 recruited	II	Mean age ≈ 68 ± 9 year; ≈ 58 % female; mean BMI ≈ 29.9 kg/m²; varus/valgus distribution balanced	CT-based PSI (MyKnee®, Medacta) versus standard extramedullary/intramedullary guides for GMK Primary TKA	Mean 28.6 ± 5.2 months (≈ 2 years)	KSS (knee and function), ROM, VAS pain, WOMAC, OKS; radiological HKA, component angles, outlier rates	PSI vs CVI: HKA deviation 1.5° vs 2.2° (p < 0.001); HKA outliers > 3°: 9.6 % vs 22.2 % (p = 0.010); better femoral and tibial component accuracy; KSS-function 86.8 vs 80.9 (p = 0.017); VAS pain 1.8 vs 2.4 (p = 0.038)	Very low revision/complication incidence in both groups; no intraoperative abandonment; no significant between-group difference	CT-based PSI significantly improves mechanical alignment and component positioning; early clinical outcomes largely equivalent - authors suggest reduced outliers may translate into better long-term results.
Sood and Yasin [20]	Prospective randomized comparative study (3D-printed CT-based PSG vs CI)	100 TKAs (PSG = 50; CI = 50)	II	Mean age 63.4 ± 7.3 years; 54 % female; mean BMI 26.7 kg/m²; baseline demographics balanced between groups	Custom-made 3D printed CT-based PSG for distal femoral and tibial cuts (Scorpio NRG PS implant) vs standard intramedullary/extramedullary guides	1 year (assessments at 6 weeks, 6 months, 12 months)	HKA angle ± outliers (> 3°), operative time, WOMAC, wound events	PSI yielded nearer-neutral alignment (mean HKA 178.4° vs 176.6°, p = 0.041), fewer outliers (16 % vs 42 %, p = 0.007) and shorter surgery (60 min vs 89 min, p < 0.001); WOMAC similar at 1 year	Minor wound-healing delay (2 PSI, 3 Conv) and superficial infection (2 each); no revisions or serious events	Patient-specific guides improved coronal alignment and saved OR time without extra morbidity; functional benefit not evident at 1 year but authors expect long-term gains
Zhou et al. [21]	Prospective single-center comparative cohort (3D-printed CT-PSI vs conventional)	52 TKAs (PSI = 19; CON = 33)	II	PSI: mean age 70.2 ± 8.4 year, 7 M/12 F, BMI 24.2 ± 3.4 kg/m²; CON: mean age 68.6 ± 7.1 year, 10 M/23 F, BMI 25.4 ± 2.8 kg/m²	Custom 3D printed PSI guide plates (no femoral IM canal) vs standard intramedullary/extramedullary guides (IM canal opened)	3 months post-surgery	Coronal alignment (FMAA, LFA, HKA, FTA), intra- and post-op blood loss, hidden loss, Hb drop; clinical scores (HSS, KSS, ROM, WOMAC, VAS)	PSI cut intra-op blood loss by ~53 % (73 ± 31 mL vs 156 ± 111 mL) and total loss by ~46 % (420 ± 211 mL vs 782 ± 340 mL, p < 0.05); HKA outliers 0 % vs 18.8 %; higher post-op HSS (86 ± 8 vs 58 ± 11) and KSS (78 ± 14 vs 44 ± 15); better WOMAC and VAS	No revisions or serious adverse events reported; PSI avoided IM canal-related bleeding	3D printed PSI improved coronal alignment accuracy, greatly reduced blood loss, and yielded better early functional scores; authors recommend PSI as a safer, more precise alternative to conventional guides
Lei et al. [24]	Retrospective cohort with 1:1 PSM (CT-based 3D pre-op design + conventional tools vs standard instrumentation)	218 TKAs (Novel = 109; Conventional = 109)	III	Mean BMI 25.6 ± 3.4 kg/m²; ≈ 20 % male; age and other baseline factors balanced post-match	CT-based patient-specific 3D planning used to set femoral entry, valgus angle, tibial guide pin and rod positions; surgery otherwise identical to conventional extramedullary/intramedullary guides	12 months	Component angles (LTC, FFC, FTC, LFC), HKA and outliers/over-correction, op and tourniquet time, LOS, blood loss, VAS pain, NEW-KSS	Novel vs conventional: LTC 4.2° ± 2.6 vs 9.5° ± 3.7 (p < 0.001); LTC outliers > 3° 31.7 % vs 95.1 %; HKA outliers 17.4 % vs 40.4 % (p < 0.001); operation 62 ± 10 vs 76 ± 19 min; tourniquet 36 ± 7 vs 49 ± 13 min; VAS at 1 month 3.6 vs 4.3 (p < 0.001); no NEW-KSS difference at 6-12 months	No revisions or serious adverse events; overall complication rates low and comparable	CT-based 3D planning markedly improves tibial component alignment, reduces outliers and early pain, and shortens surgery time; functional outcomes equal by 1 year, but authors see potential long-term benefit
DeHaan et al. [25]	Single-surgeon retrospective cohort (MRI-based Visionaire™ PSI vs conventional)	356 TKAs (PSI = 306; Conventional = 50)	III	Mean age 62.8 ± 10.3 year; 63.7 % female (PSI 68 %, Conventional 38 %); mean BMI 32.2 ± 6.6 kg/m²	Smith & Nephew Visionaire™ MRI-based custom cutting blocks for femur and tibia vs standard intramedullary/extramedullary guides (Legion/Journey implants)	Perioperative period to hospital discharge (median POD 3)	Tourniquet and total operative time, OR turnover, intra-op blood loss, Hb/Hct drop, drain output, transfusion, LOS, sizing accuracy, cost	PSI vs Conventional: operative time ↓ 20.4 min (86.8 ± 16.9 vs 107.2 ± 29.5; p < 0.01); tourniquet ↓ 19.9 min (63.3 ± 16.8 vs 83.2 ± 22.2; p = 0.024); OR turnover ↓ 42 % (15.2 ± 7.8 vs 21.6 ± 6.5 min; p = 0.022); no differences in blood loss, Hb/Hct change, drain output, transfusion, or LOS; sizing accurate in 81 % femur and 90 % tibia; cost savings from time and tray reduction (~ US$ 1,566) offset imaging + jigs (US$ 830-1,860)	No intraoperative complications; transfusion 4 PSI vs 1 Conventional (ns); overall peri-op morbidity equivalent	PSI shortens surgery and turnover without increasing morbidity and is cost-neutral or cost-saving; implant sizing highly reliable, supporting routine PSI use in primary TKA
Steimle et al. [26]	Retrospective cohort (simultaneous bilateral TKA: MRI-PSI vs conventional)	80 bilateral TKA patients (PSI = 31; Conventional = 49)	III	Mean age ≈ 61 years; mean BMI ≈ 34 kg/m²; ASA 2-3; comorbidities and demographics comparable between groups	Visionaire™ MRI-based patient-specific cutting jigs (Zimmer Persona/Natural PS implants) vs standard intramedullary/extramedullary guides (off-the-shelf Stryker Triathlon/Zimmer)	Immediate perioperative period to discharge (48 h outcomes, length of stay)	Surgical and tourniquet time, intra-op blood loss, Hb drop, transfusion needs, opioid use (MME), LOS, discharge disposition	No significant differences: surgery time ≈ 102 vs 99 min; tourniquet ≈ 63 vs 60 min; blood loss ≈ 275 vs 254 mL; Hb drop, transfusion rates, opioid use, and LOS (4.4 vs 4.5 days) all similar	No major perioperative complications in either group; transfusion requirements comparable	PSI offered no immediate perioperative advantages over CI in bilateral TKA; authors conclude PSI is not beneficial for acute outcomes
Sun et al. [27]	Prospective RCT (3D-printed patient-specific IM guide vs conventional)	80 TKAs (PSI = 40; CON = 40)	II	Mean age ≈ 68 years; 65 F/15 M; mean BMI ≈ 26 kg/m²; groups demographically matched	Novel 3D-printed PSI for femoral entry and rotation (LEGION PS implant) vs standard intramedullary/extramedullary guides	Mean 9 ± 4 months (range 7-12)	Operation time, drainage volume/duration, HSS and AKS scores, ROM; radiological HKA, PFA, PCA, depth of IM guide	PSI ↓ drainage (259 ± 12 mL vs 306 ± 11 mL) but ↑ op time (87 ± 4 min vs 74 ± 4 min); more accurate rotation: PFA 0.5° ± 0.3 vs 3.1° ± 1.0; PCA 0.4° ± 0.2 vs 1.7° ± 2.0; IM-guide depth 9.4 ± 2.5 cm vs 20.4 ± 3.6 cm; no HSS/AKS difference at 6 months	No intra-op or early post-op complications reported in either group	PSI improves femoral rotational alignment and reduces blood loss with similar short-term clinical scores, albeit at the cost of ~15 min extra OR time; authors support PSI for greater accuracy
Barrett et al. [28]	Prospective multicenter non-randomized study (CT-based TruMatch™ PSI vs conventional and CAS)	66 PSI TKAs; historical controls: Conventional = 86, CAS = 81	III	62 % female; mean age 66 ± 8 year; mean BMI 33 ± 7 kg/m²; 99 % osteoarthritis	TruMatch CT-based patient-specific femoral/tibial cutting blocks (PFC Sigma implants) vs standard intramedullary/extramedullary guides or imageless computer navigation	≈ 12 weeks (long-leg radiographs at 11 weeks; clinical follow-up mean 87 days)	Absolute mechanical-axis deviation, femoral/tibial component angles, % within ±3°, skin-to-skin and tourniquet time, adverse events	PSI absolute deviation 2.23° vs 2.09° (conventional) and 1.94° (CAS); 81 % within ±3° vs 77 % and 83 %; skin-to-skin 74.6 min vs 79.8 min (conv., ns) and 101.2 min (CAS, p < 0.001); tourniquet 61.7 min vs 60.5 min (conv.) and 82.5 min (CAS, p < 0.001); smaller SDs for component alignment and time with PSI	One case of arthrofibrosis; no other serious events; overall morbidity comparable across groups	CT-based PSI yields mechanical alignment non-inferior to conventional/CAS, trims OR time versus CAS, and reduces variability, supporting PSI as an efficient, cost-neutral alternative to standard techniques
Liao et al. [29]	Prospective RCT (AI-driven CT-based intelligent PSI vs conventional)	107 TKAs (i-PSI = 54; CI = 53)	II	Mean age ≈ 68 ± 7 years; ≈ 20 % male; mean BMI ≈ 26 kg/m²; groups demographically balanced	AI-generated, 3D-printed intelligent PSI guides for distal femur and proximal tibia (Attune PS implant) vs standard intramedullary/extramedullary guides	Early postoperative (perioperative period to discharge; radiographs/CT within days)	Resection accuracy (deviation and ratio), coronal and sagittal alignment (HKA, FCA, TCA, JLCA, FSA, TSA, PCA), operative time, blood and Hb loss, complications	i-PSI cut distal femur more accurately (lat −0.38 mm vs −0.72 mm; med −0.21 mm vs −0.58 mm; p ≈ 0.03); improved HKA (0.6° vs 1.6° varus, p = 0.025) with fewer outliers (11 % vs 26 %); better FCA, JLCA, FSA; operative time ↑ ≈ 6 min (82 vs 76 min, p = 0.027); blood loss and Hb drop similar	One superficial wound-healing issue in CI group; no additional adverse events; overall complication rates comparable	AI-based i-PSI simplifies production, enhances resection precision and yields more neutral limb/component alignment without added morbidity or blood loss, supporting its clinical adoption.
Giannotti et al. [30]	Single-center RCT (CT-based single-use PSI vs conventional)	40 TKAs (PSI = 20; Control = 20)	II	PSI: mean age 71 years, 4 M/16 F; Control: mean age 73 years, 10 M/10 F; all primary OA	Medacta GMK Efficiency® CT-based, single-use patient-specific guides vs standard reusable Medacta GMK Primary instruments	Radiographs at 1 month; clinical review at 2 months	KSS, total blood loss, femorotibial angle (FTA), mechanical component	PSI cut blood loss by ≈ 24 % (657 mL vs 866 mL, p < 0.05); no significant differences in KSS (85.2 vs 87.2) or FTA (178.8° vs 178.9°)	No early complications in either group (no infections, implant failures, vascular/nerve injury)	PSI meaningfully reduces perioperative blood loss but offers no alignment or early functional advantage; authors recommend its selective use when minimal blood loss or difficult femoral cuts are anticipated.
Pauzenberger et al. [31]	Retrospective cohort (CT-based MyKnee® PSI vs conventional)	1,257 TKAs (CVI = 442; PSI = 815)	III	Mean age ≈ 69 years; ≈ 65 % female; pre-operative varus knees ≈ 71 % vs 70 %; baseline characteristics broadly comparable	CT-based MyKnee® 3D-printed cutting blocks (Medacta GMK Primary) vs standard extramedullary tibial and intramedullary femoral instrumentation	Immediate post-op radiographs / CT (in-hospital)	HKA deviation and outliers (> 3°); component angles (FFC, FTC, LFC, LTC); femoral component rotation (FCR); severe outliers (> 5°)	PSI vs CVI: mean HKA deviation 1.7° ± 1.2 vs 2.3° ± 1.7 (p < 0.001); HKA outliers 10.1 % vs 25.8 %; FCR outliers 3.2 % vs 39.2 %; severe outliers virtually eliminated; absolute deviations smaller for all planes	No surgery-related or early post-op complications reported (radiographic study only)	CT-based PSI significantly improves mechanical axis restoration and 3D component positioning, preventing severe malalignment; authors recommend PSI to enhance accuracy
Qui et al. [32]	Prospective comparative cohort (CT-based 3D-printed PSI vs conventional)	26 TKAs (PSI = 10; CI = 16)	II	Mean age ≈ 67.6 ± 7 year (PSI) vs 65.5 ± 6.6 year (CI); sex PSI 4 M/6 F, CI 2 M/14 F; laterality balanced; pre-op coronal mal-alignment ≈ 8.9° varus in both groups	CT-based 3D imaging, virtual planning and custom-printed distal-femur / proximal-tibia cutting guides vs standard intramedullary (femur) and extramedullary (tibia) guides	Immediate postoperative radiographs / CT (in-hospital)	Execution accuracy (cut depth/angles), post-op HKA, FFC, FTC, LTC angles, outliers > 3°	PSI vs CI: HKA deviation 0.77 ± 0.48° vs 3.13 ± 1.78°; FFC 0.37 ± 0.51° vs 2.35 ± 2.51°; FTC 0.11 ± 0.23° vs 1.09 ± 1.58°; LTC 0.71 ± 0.73° vs 0.66 ± 1.74° (all p < 0.05 except LTC); outliers (> 3°) for HKA 0 % vs 44 %, FFC 0 % vs 44 %, FTC 0 % vs 6 %; intra-op cut parameter deviations < 1 mm / 0.5° with PSI	No intraoperative or early post-op complications reported in either group	3D-printed PSI executed the pre-op plan with sub-millimeter/degree accuracy, virtually eliminated alignment outliers, and avoided intramedullary instrumentation - all without added morbidity; authors advocate PSI to enhance precision in primary TKA
Gemalmaz et al. [33]	Prospective quasi-randomized cohort (CT-based PSI vs conventional)	40 TKRs (PSI = 20; Conventional = 20)	III	PSI group: 5 M/15 F; mean age 68.6 ± 8.6 year. Control: 4 M/16 F; mean age 70.4 ± 7.1 year. Baseline alignment comparable.	Custom CT-derived cutting blocks for distal femur and tibia (NexGen CR-Flex implant) vs standard intramedullary femoral and extramedullary tibial instrumentation	Immediate post-op long-leg radiographs (in-hospital)	Post-op mFTA, FCA, TCA; frequency of outliers > 3° for limb axis or components	Mean mFTA: 2.09° ± 1.27 (PSI) vs 2.84° ± 2.19 (ns). Outliers > 3° mFTA: 5 % vs 35 % (p = 0.04). Component outliers > 3°: 5 % vs 27.5 % (p = 0.006). No sig. Differences in FCA or TCA means.	No intraoperative or early post-op adverse events reported in either group	PSI did not change mean alignment values but significantly reduced the proportion of malalignment outliers, supporting PSI use to improve accuracy in primary TKA.
Yamamura et al. [34]	Case-control cohort comparing two CT-based PSI designs	60 TKAs (Conventional PSI = 30; New PSI = 30)	III	Mean age ≈ 70 years; 77 %-87 % female; mean BMI ≈ 26.7 kg/m²; all varus OA knees; groups demographically matched	Newly designed Prophecy® CT-based PSI (larger bone-contact area, rotational marker, longer extramedullary-rod channel, AP marker pin) vs conventional Prophecy® PSI for Evolution PS implant	Early post-op: 3D CT at 2 weeks	Absolute difference between pre-op plan and post-op tibial alignment (coronal, sagittal, axial); deviation > 3° in each plane	New vs conventional PSI: coronal deviation 1.2 ° ± 0.8 vs 2.0 ° ± 1.6 (p = 0.045); axial 2.6 ° ± 1.6 vs 6.2 ° ± 5.5 (p = 0.004); sagittal ns. Deviations > 3°: coronal 0 % vs 27 %, sagittal 20 % vs 30 % (ns), axial 20 % vs 63 % (p = 0.001)	No intraoperative or early postoperative adverse events reported	Design modifications significantly improved tibial component accuracy in coronal and axial planes and cut deviation rates without added morbidity - authors recommend further PSI design optimization

Quality Assessment of Included Studies

Fourteen papers were evaluated using the modified Downs and Black Checklist (maximum score = 28). The median total score was 19 (IQR 18-22). Reporting quality and protection against measurement bias were consistently strong, while external validity and control of confounders were more variable, particularly in retrospective cohorts.

Applying standard cut-offs (Excellent ≥26; Good 21-25; Fair 15-20; Poor ≤14), five studies were classified as Good and nine as Fair; none were rated Excellent or Poor. This indicates an evidence base of acceptable quality, although broader multicenter sampling and more rigorous confounder adjustment would strengthen future research. Table 3 summarizes the quality assessment scores and domain-specific ratings for each included study.

**Table 3:** Quality assessment of the 14 included studies using the Modified Downs and Black Checklist Scores are reported for each of the five domains and summed to a maximum of 28. Quality categories are defined as Excellent (≥ 26), Good (21-25), Fair (15-20), and Poor (≤ 14).

Study (citation)	Study design	Reporting (0-11)	External validity (0-3)	Internal validity - bias (0-7)	Internal validity - confounding (0-6)	Power (0-1)	Total /28	Quality rating
Anderl et al. [19]	Prospective comparative cohort	8	2	5	4	1	20	Good
Sood and Yasin [20]	Prospective randomized trial	9	2	6	5	1	23	Good
Zhou et al. [21]	Prospective comparative cohort	8	2	5	4	0	19	Fair
Lei et al. [24]	Retrospective PSM cohort	8	2	4	4	0	18	Fair
DeHaan et al. [25]	Retrospective cohort	8	2	4	4	0	18	Fair
Steimle et al. [26]	Retrospective bilateral-TKA cohort	7	2	4	4	0	17	Fair
Sun et al. [27]	Randomized controlled trial	9	2	6	4	1	22	Good
Barrett et al. [28]	Multicenter non-randomized study	8	3	5	3	0	19	Fair
Liao et al. [29]	Randomized controlled trial	9	2	6	6	1	24	Good
Giannotti et al. [30]	Randomized clinical trial	9	2	5	4	1	21	Good
Pauzenberger et al. [31]	Retrospective radiographic cohort	8	2	4	4	0	18	Fair
Qiu et al. [32]	Prospective comparative cohort	8	2	4	4	0	18	Fair
Gemalmaz et al. [33]	Prospective quasi-randomized cohort	8	2	4	4	0	18	Fair
Yamamura et al. [34]	Case-control PSI design study	8	2	4	4	0	18	Fair

Results of Meta-Analysis

**Figure 2:**
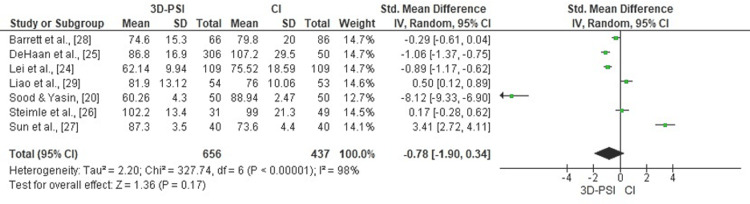
Forest plot comparing 3D-PSI with CI for surgical time in TKA The plot illustrates the pooled SMDs with 95% CIs across included studies. Negative values favor 3D-PSI, indicating reduced surgical time compared to CI. Study weights were calculated using a random-effects model. 3D-PSI, 3D patient-specific instrumentation; CI, conventional instrumentation; SMD, standardized mean difference; TKA, total knee arthroplasty Sources: [20,24-29]

Surgical time: Meta-analysis of seven studies revealed no statistically significant difference in surgical time between 3D-PSI and CI in TKA (pooled SMD = -0.78, 95% CI: -1.90 to 0.34, p = 0.17). Considerable heterogeneity was observed among studies (χ² = 327.74, df = 6, p < 0.00001; I² = 98%), indicating substantial variation in reported effect sizes. The corresponding forest plot is shown in Figure 2.

**Figure 3:**
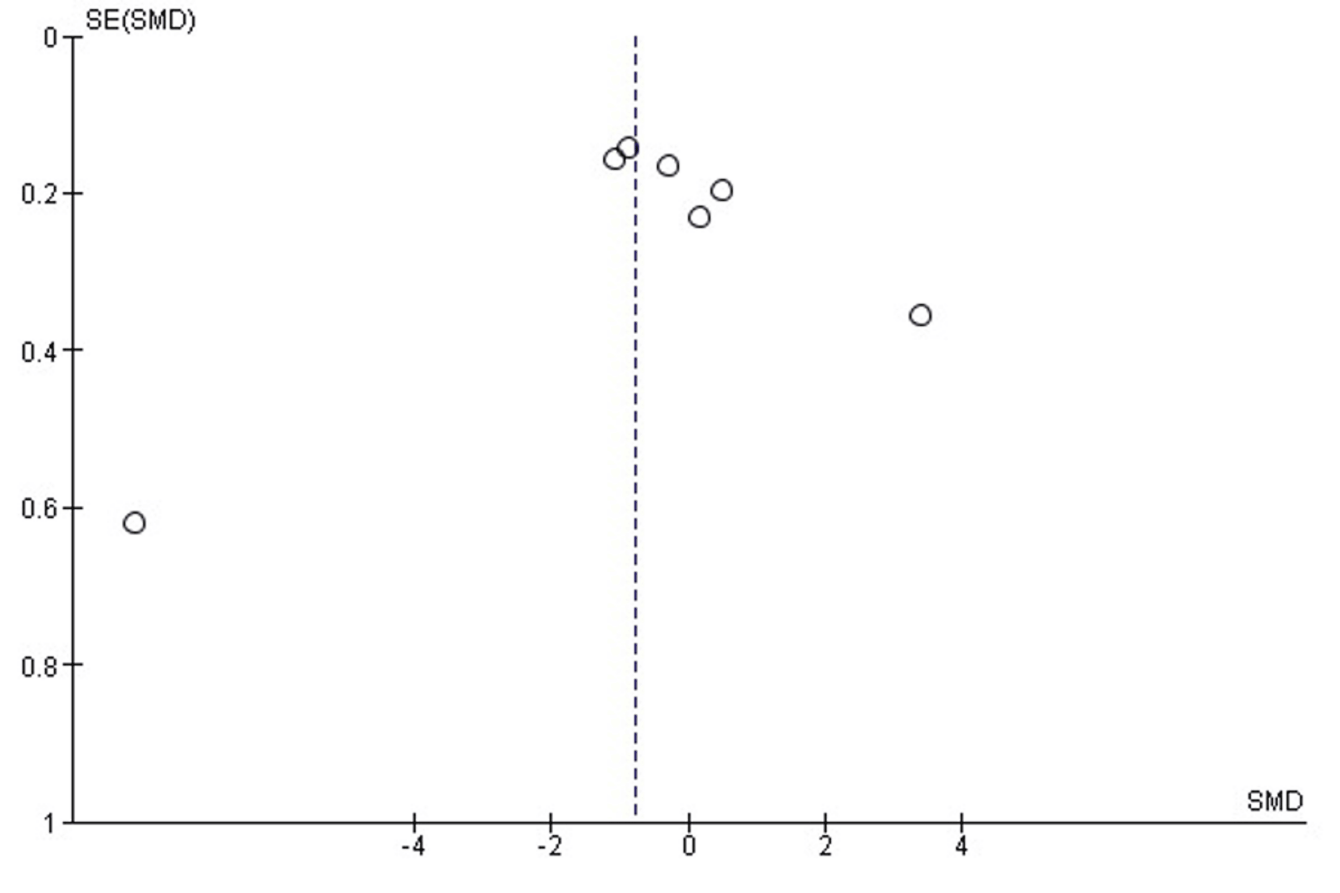
Funnel plot assessing publication bias in studies comparing 3D-PSI and CI for surgical time in TKA The plot displays the distribution of effect sizes against their standard errors. A symmetrical inverted funnel shape suggests the absence of significant publication bias, while visible asymmetry may indicate potential small-study effects. 3D-PSI, 3D patient-specific instrumentation; CI, conventional instrumentation; SMD, standardized mean difference; TKA, total knee arthroplasty

Publication bias assessment for surgical time: Visual inspection of the funnel plot (Figure 3) revealed some asymmetry, suggesting potential publication bias or small-study effects. However, Egger’s regression test was not statistically significant (p > 0.05), indicating no strong evidence of publication bias.

**Figure 4:**
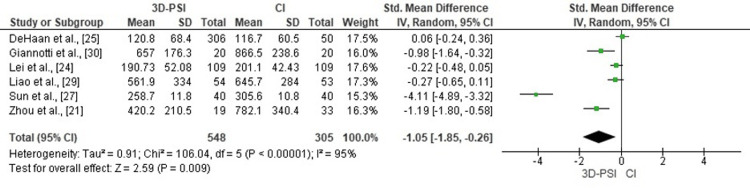
Forest plot comparing 3D-PSI with CI for intraoperative blood loss in TKA The pooled analysis demonstrates a significant reduction in blood loss with 3D-PSI. Each horizontal line represents an individual study with its 95% CI, while the diamond represents the overall effect estimate. 3D-PSI, 3D patient-specific instrumentation; CI, conventional instrumentation; TKA, total knee arthroplasty Sources: [21,24,25,27,29,30]

Blood loss: Meta-analysis of six studies demonstrated that 3D-PSI significantly reduced intraoperative blood loss compared with CI in TKA (pooled SMD = -1.05, 95% CI: -1.85 to -0.26, p = 0.009). High heterogeneity was observed among studies (χ² = 106.04, df = 5, p < 0.00001; I² = 95%), indicating substantial variation in effect sizes across datasets. The corresponding forest plot is shown in Figure 4.

**Figure 5:**
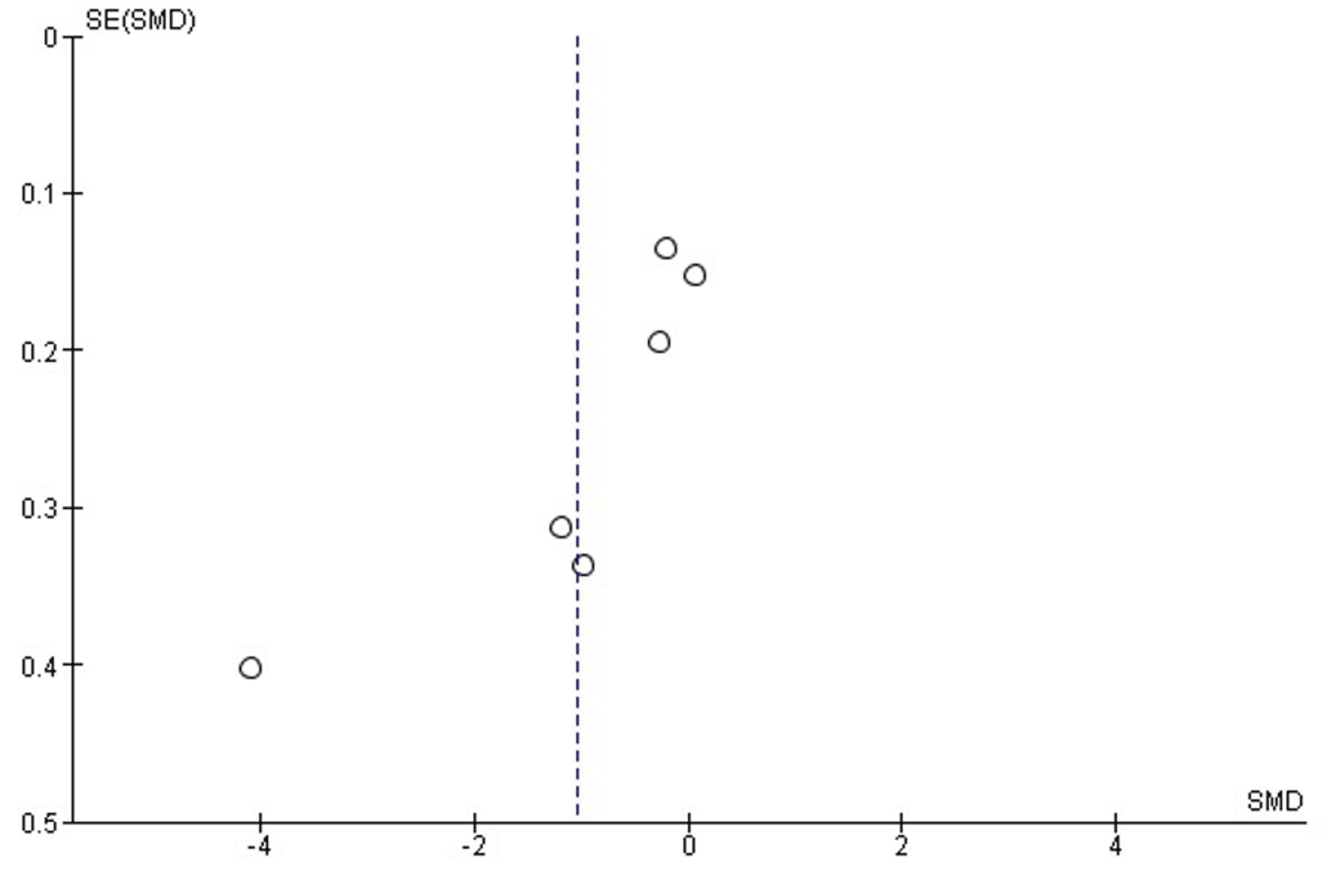
Funnel plot assessing publication bias in studies comparing 3D-PSI and CI for blood loss in TKA The plot exhibited some asymmetry, with one or two studies deviating from the rest, suggesting potential publication bias. However, Egger’s regression test was not statistically significant (p > 0.05), indicating no strong evidence of small-study effects or publication bias. 3D-PSI, 3D patient-specific instrumentation; CI, conventional instrumentation; SMD, standardized mean difference; TKA, total knee arthroplasty

Publication bias assessment for blood loss: The funnel plot for blood loss exhibited some asymmetry, with one or two studies markedly deviating from the rest, suggesting potential publication bias. However, Egger’s regression test was not statistically significant (p > 0.05), indicating no strong evidence of small-study effects or publication bias (Figure 5).

**Figure 6:**
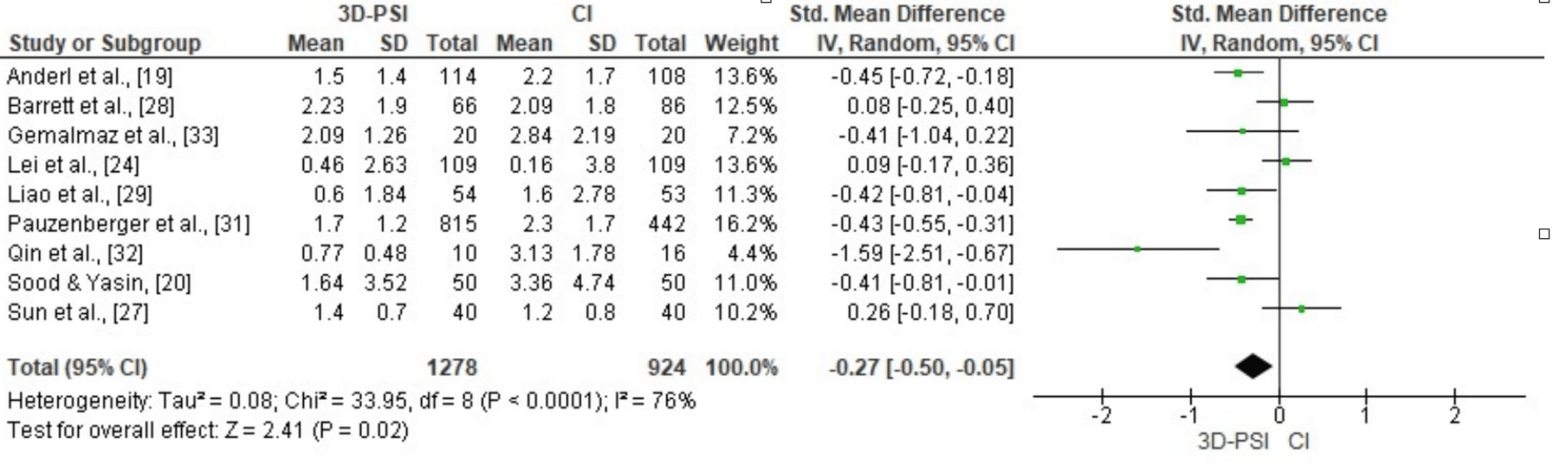
Forest plot comparing 3D-PSI with CI for HKA alignment accuracy in TKA The forest plot displays the pooled SMD with 95% CIs for alignment accuracy across nine studies. Negative values favor PSI, indicating reduced deviation from the neutral HKA axis compared to CI. 3D-PSI, 3D patient-specific instrumentation; CI, conventional instrumentation; HKA, hip-knee-ankle; TKA, total knee arthroplasty Sources: [19,20,24,27-29,31-33]

HKA alignment accuracy: Meta-analysis of nine studies comparing the mean absolute deviation from the neutral HKA angle demonstrated that 3D-PSI significantly improved alignment accuracy compared with CI (pooled SMD = -0.27, 95% CI: -0.50 to -0.05, p = 0.02). The analysis showed moderate to substantial heterogeneity (χ² = 33.95, df = 8, p < 0.0001; I² = 76%), indicating variability in outcomes among the included studies (Figure 6).

Although these findings confirm greater radiographic precision with PSI, their long-term clinical relevance remains uncertain. Minor improvements in alignment may not necessarily translate into measurable benefits in pain relief, functional recovery, or implant survival, as these outcomes are influenced by multiple factors such as soft-tissue balance, implant design, and postoperative rehabilitation. Current evidence, therefore, suggests that while accurate alignment is desirable, its contribution to long-term clinical outcomes appears limited.

**Figure 7:**
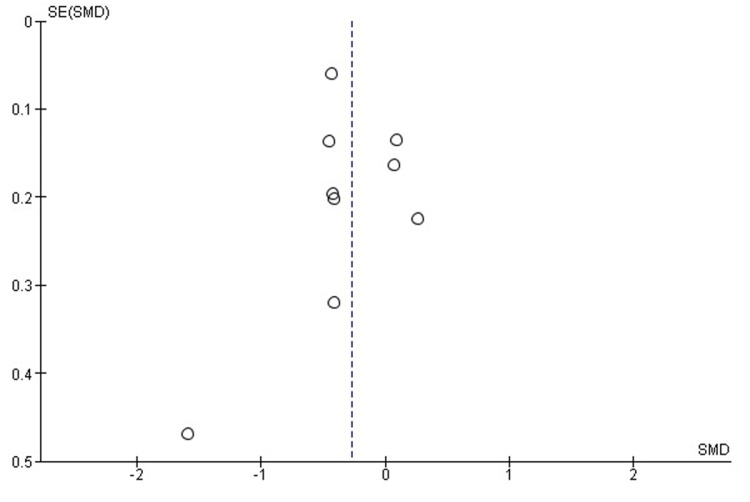
Funnel plot assessing publication bias in studies comparing 3D-PSI and CI for HKA alignment accuracy in TKA The funnel plot illustrates the distribution of included studies for HKA alignment accuracy. The symmetrical scatter of effect estimates around the pooled mean indicates an absence of significant publication bias. Egger’s test confirmed this with a non-significant result (p > 0.05). 3D-PSI, 3D patient-specific instrumentation; CI, conventional instrumentation; HKA, hip-knee-ankle; SMD, standardized mean difference; TKA, total knee arthroplasty

Publication bias assessment for HKA alignment accuracy: Visual inspection of the funnel plot for HKA alignment accuracy revealed a relatively symmetrical distribution of studies around the pooled effect size, suggesting no major publication bias. This finding was supported by Egger’s regression test, which was not statistically significant (p > 0.05) (Figure 7).

**Figure 8:**
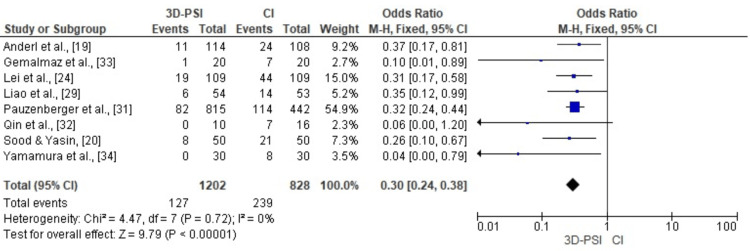
Forest plot comparing 3D-PSI with CI for the rate of HKA alignment outliers (>±3°) in TKA The forest plot demonstrates a significantly lower incidence of HKA outliers in the 3D-PSI group compared to CI. The narrow CIs and absence of heterogeneity (I² = 0%) indicate robust and consistent results across included studies. 3D-PSI, 3D patient-specific instrumentation; CI, conventional instrumentation; HKA, hip-knee-ankle; TKA, total knee arthroplasty Sources: [19,20,24,29,31-34]

HKA outlier rates: Meta-analysis of eight studies demonstrated that 3D-PSI significantly reduced the odds of postoperative HKA alignment outliers (defined as >±3° deviation) compared to CI (pooled OR = 0.30, 95% CI: 0.24 to 0.38, p < 0.00001). The analysis showed no significant heterogeneity (χ² = 4.47, df = 7, p = 0.72; I² = 0%), indicating consistent findings across studies. The corresponding forest plot is shown in Figure 8.

**Figure 9:**
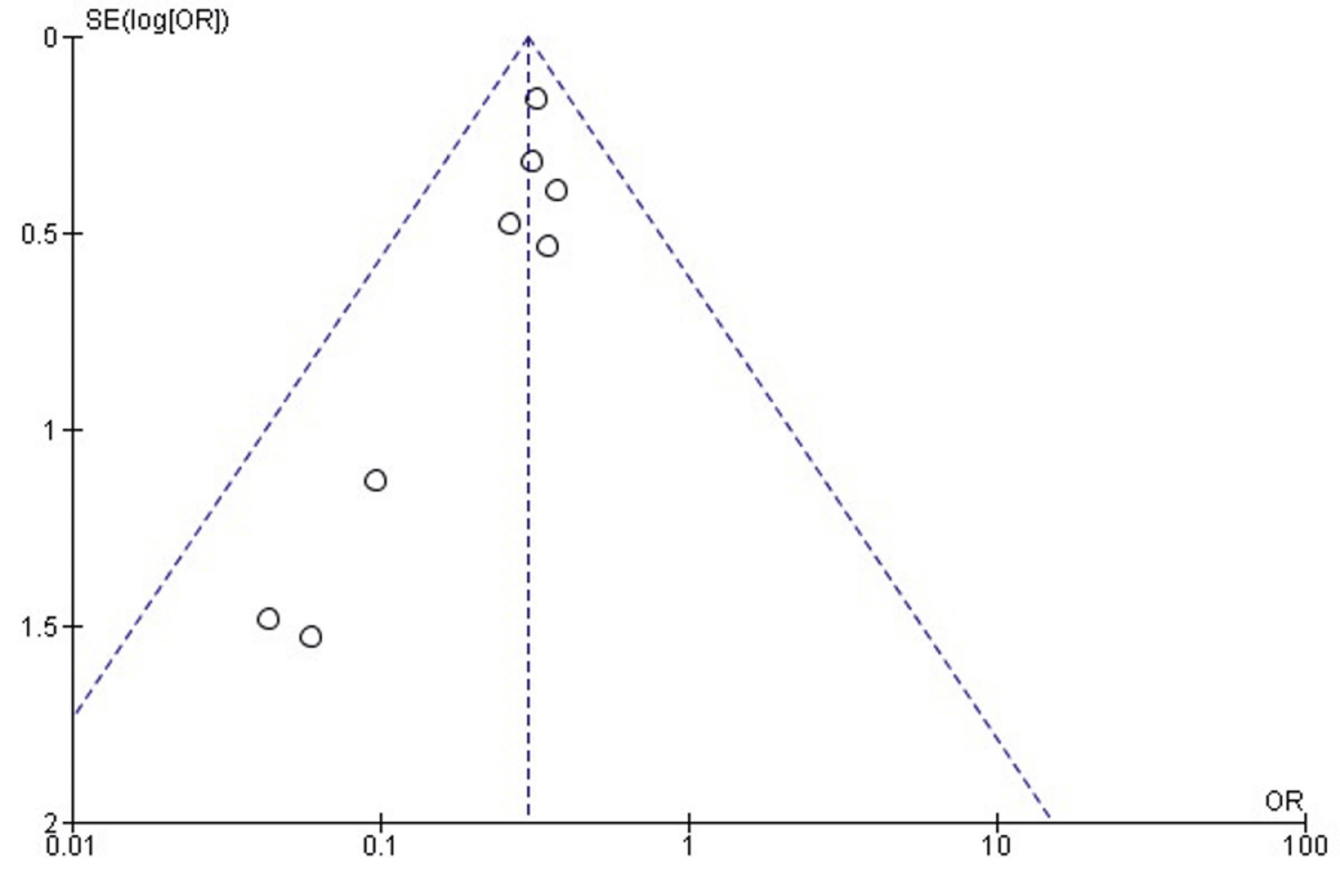
Funnel plot assessing publication bias in studies comparing 3D-PSI and CI for HKA outlier rates in TKA The funnel plot shows a symmetrical distribution of studies around the pooled estimate, indicating no major evidence of publication bias or small-study effects for HKA outlier outcomes. 3D-PSI, 3D patient-specific instrumentation; CI, conventional instrumentation; HKA, hip-knee-ankle; SMD, standardized mean difference; TKA, total knee arthroplasty

Publication bias assessment for HKA outlier rates: The funnel plot for HKA outlier rates shows a reasonably symmetrical distribution of study effects, suggesting the absence of significant publication bias. This is further supported by Egger’s regression test, which was not statistically significant (p > 0.05) (Figure 9).

Discussion

This systematic review and meta-analysis compared the effectiveness of 3D-PSI with CI in TKA, evaluating outcomes such as alignment accuracy, surgical time, intraoperative blood loss, and malalignment outlier rates. Fourteen studies comprising 2,704 procedures were included, representing diverse patient populations, implant systems, and geographic regions. These findings contribute to the growing body of evidence on how technological innovations can enhance surgical precision and outcomes in joint replacement.

Alignment Accuracy

The most consistent benefit observed with 3D-PSI was a significant reduction in alignment outliers. Pooled analysis of eight studies revealed that patients in the PSI group were approximately 70% less likely to experience malalignment greater than ±3° from the neutral mechanical axis compared with those treated with conventional guides (OR = 0.30, p < 0.00001). This aligns with findings from Anderl et al. [19], Sood and Yasin [20], and Pauzenberger et al. [31], all demonstrating more accurate reproduction of planned alignment and component positioning with personalized guides. Improved alignment is clinically relevant, as it is associated with implant longevity and a reduced risk of premature failure. Mechanisms contributing to this benefit include patient-specific contouring of cutting blocks, stable guide fixation, and elimination of femoral intramedullary instrumentation, which can introduce variability.

Intraoperative Blood Loss

Intraoperative blood loss was another area of clear advantage for PSI. Meta-analysis of six studies showed a significant reduction in blood loss for the PSI group (SMD = -1.05, p = 0.009), with studies such as Zhou et al. [21] and Giannotti et al. [30] reporting reductions of up to 40-50%. This finding is clinically meaningful, particularly in elderly or anemic patients and in settings where blood conservation is critical. The non-intramedullary design of PSI likely contributes to reduced bleeding by avoiding canal pressurization and limiting marrow exposure. Additionally, shorter operative durations reported in some studies suggest a more efficient surgical process that further minimizes tissue trauma and blood loss.

Surgical Time and Efficiency

Although several studies have reported operative efficiency gains with PSI, including shorter surgical times and fewer instrument trays, our meta-analysis did not identify a statistically significant difference in overall surgical duration (SMD = -0.78, p = 0.17). The considerable heterogeneity observed in surgical time likely reflects multiple contributing factors. Variations in surgeon experience, learning curves, and familiarity with PSI workflows can markedly affect operative efficiency, particularly during the early adoption phase of 3D-printed guide implementation. Institutional infrastructure and operating room logistics, such as preoperative planning time, guide sterilization protocols, and team coordination, also play critical roles. Furthermore, differences in surgical approach (minimally invasive versus standard medial parapatellar) and case complexity may further explain time discrepancies across centers. These contextual factors indicate that operative time outcomes should be interpreted with caution, as they are highly dependent on local expertise and system integration rather than the instrumentation technique alone. For example, DeHaan et al. [25] reported a 20-minute reduction in operating time and notable perioperative cost savings in a high-volume center, whereas Sun et al. [27] observed longer procedures due to additional guide positioning and verification steps. Such contrasting findings emphasize the context-dependent nature of PSI efficiency, with potential benefits more pronounced in standardized, high-throughput surgical environments.

Functional Outcomes

Although PSI improves radiographic alignment and reduces intraoperative blood loss, these technical advantages have not consistently translated into superior long-term functional outcomes. While some studies reported better early KSSs or reduced pain with PSI, most, including Steimle et al. [26] and Liao et al. [29], found comparable functional recovery between PSI and CI at 6-12 months. Similarly, several large-scale randomized controlled trials have failed to demonstrate significant long-term functional differences between PSI and CI despite clear short-term radiographic advantages. This inconsistency likely reflects the multifactorial nature of postoperative functional recovery, which is influenced by implant design, soft-tissue balance, and rehabilitation quality rather than alignment alone.

Cost-Effectiveness and Clinical Implications

The cost-effectiveness of PSI remains context-dependent. Potential savings from reduced blood loss, fewer instrument trays, and shorter operative times may offer advantages in high-volume centers with streamlined workflows. However, these benefits are less pronounced in low-volume or resource-limited settings, where the cost of preoperative imaging, guide production, and logistical coordination is distributed across fewer cases. As such, PSI is likely to be most economically viable when implemented within standardized, high-throughput systems or selectively in complex cases where CI is less effective. Further economic modeling is warranted to clarify its overall value across different healthcare settings. While PSI enhances surgical precision and component positioning, it does not directly alter soft-tissue dynamics or patient biomechanics, key determinants of sustained clinical outcomes. Consequently, radiographic improvements may not always translate into measurable long-term functional benefits. This suggests that short-term technical precision does not necessarily correspond to patient-perceived improvements, particularly when postoperative rehabilitation and implant designs are standardized. These findings also highlight the need to consider the cost-effectiveness of PSI when improved alignment does not yield demonstrable functional or survivorship gains.

Applications in Complex and Revision Cases

PSI may offer distinct advantages in select patient populations. In individuals with complex deformities or altered anatomy following trauma, PSI enables detailed preoperative planning and guides customization beyond the capabilities of conventional instruments. Studies such as Qiu et al. [32] and Yamamura et al. [34] demonstrated that PSI can achieve greater coronal and axial alignment precision in challenging knees, potentially reducing the risk of component malrotation, a known cause of postoperative instability and pain. From a systems perspective, PSI may also streamline operating room logistics. DeHaan et al. [25] documented reductions in the number of instrument trays, shorter sterilization cycles, and reduced handling, resulting in potential cost savings. These efficiencies were particularly evident in centers with centralized planning and manufacturing capabilities, where preoperative imaging and guide production costs were offset by intraoperative time savings.

Safety Profile

Importantly, the safety profile of PSI was comparable to CI. Across all studies, there were no reports of increased complication rates, intraoperative errors, or revisions attributable to PSI. Zhou et al. [21] and Gemalmaz et al. [33] reported fewer instances of intraoperative bleeding and improved early range of motion in PSI groups, supporting the conclusion that PSI is both effective and safe when applied by experienced surgeons following validated protocols.

Limitations

This review has several limitations. Most included studies were observational, which introduces potential selection bias and residual confounding that may influence pooled outcomes. Only a minority were randomized controlled trials, and few reported long-term follow-up, limiting conclusions regarding implant survival or late functional performance. Furthermore, heterogeneity in imaging modalities, guide design, and surgical technique further restricts direct comparability. There was also variability in how functional outcomes were measured across studies, particularly in the versions and timing of KSS and WOMAC assessments, which may have introduced measurement bias and contributed to heterogeneity. These factors highlight that while the findings support the technical advantages of 3D-PSI, the strength of evidence remains moderate, and longer-term, high-quality randomized studies are needed to confirm durability and cost-effectiveness.

## Conclusions

This meta-analysis demonstrates that 3D-PSI offers measurable technical advantages over CI in TKA, particularly in terms of improved alignment accuracy, fewer malalignment outliers, and reduced intraoperative blood loss. However, long-term functional outcomes and operative times appear comparable between techniques, suggesting that PSI may be most beneficial in anatomically complex cases or in high-volume centers where workflow efficiency and radiographic precision are priorities.

The current evidence remains limited by heterogeneity in study design, relatively short follow-up durations, and the small number of high-quality randomized trials. Therefore, while PSI shows promise as a valuable adjunct to conventional methods, further large-scale, multicenter randomized controlled trials are warranted to assess its cost-effectiveness, implant survivorship, and long-term functional outcomes. Future studies should also include extended follow-up and incorporate patient-reported outcome measures to evaluate satisfaction, pain, and quality of life, thereby improving the generalizability and clinical applicability of the findings.
